# Modeling Zinc Complexes Using Neural Networks

**DOI:** 10.1021/acs.jcim.4c00095

**Published:** 2024-04-08

**Authors:** Hongni Jin, Kenneth M. Merz

**Affiliations:** † Department of Chemistry, Michigan State University, East Lansing, Michigan 48824, United States; ‡ Department of Biochemistry and Molecular Biology, Michigan State University, East Lansing, Michigan 48824, United States

## Abstract

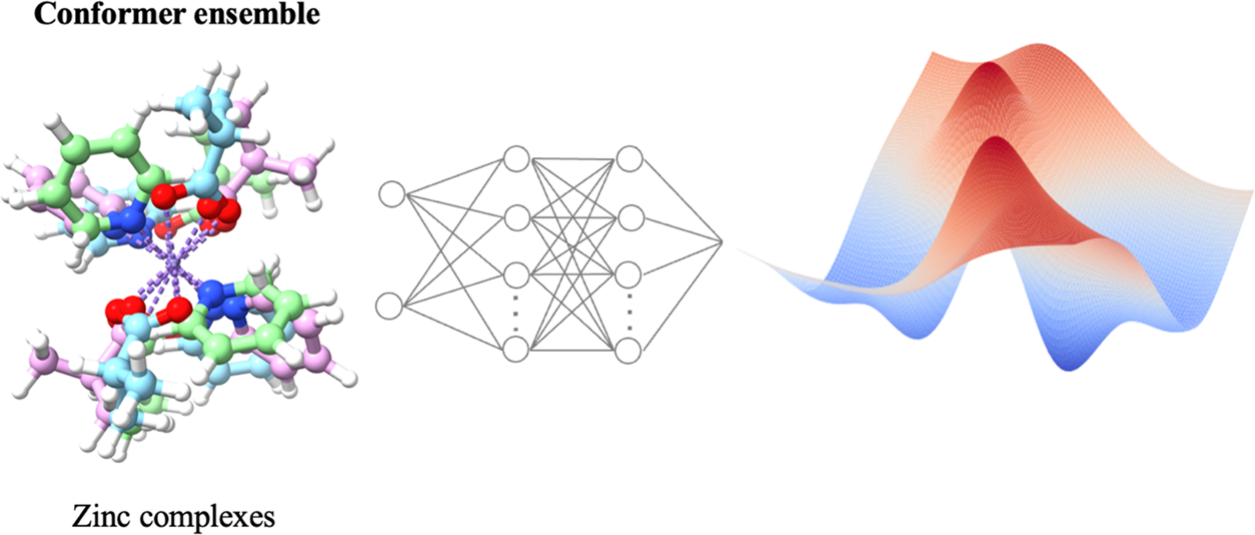

Understanding the energetic landscapes of large molecules is necessary for the study of chemical and biological systems. Recently, deep learning has greatly accelerated the development of models based on quantum chemistry, making it possible to build potential energy surfaces and explore chemical space. However, most of this work has focused on organic molecules due to the simplicity of their electronic structures as well as the availability of data sets. In this work, we build a deep learning architecture to model the energetics of zinc organometallic complexes. To achieve this, we have compiled a configurationally and conformationally diverse data set of zinc complexes using metadynamics to overcome the limitations of traditional sampling methods. In terms of the neural network potentials, our results indicate that for zinc complexes, partial charges play an important role in modeling the long-range interactions with a neural network. Our developed model outperforms semiempirical methods in predicting the relative energy of zinc conformers, yielding a mean absolute error (MAE) of 1.32 kcal/mol with reference to the double-hybrid PWPB95 method.

## Introduction

Zinc is an essential element for all living things on earth.^1−4^ Its biological function derives from its diverse structural and catalytic roles over thousands of enzymes.^5−9^ It exists in ∼3000 human proteins and participates in DNA synthesis, signal transduction, immune cell function, etc.^10,11^ Different types of zinc complexes have demonstrated potential in medical applications, such as acting as an anti-Alzheimer’s agent,^12^ anticonvulsant,^13^ and antidiabetic treatment.^14−16^

Zinc, with an [Ar]3d^10^4s^2^ electronic configuration, has only one oxidation state Zn^2+^ that has a completely filled d-shell. Such a configuration enables Zn^2+^ to accommodate various ligands in a highly flexible coordination geometry and to have relatively fast ligand-exchange reactions.^17^ Zinc usually coordinates with N, O, and S donor ligands in tetrahedral, trigonal bipyramidal, or octahedral geometries. One type of classical force field method for zinc-protein modeling is the zinc AMBER force field (ZAFF),^18^ which focuses on simulating the tetrahedral zinc complexes based on the bonded model as well as the electrostatics model. Later, Yu extended the ZAFF method to a wider range of coordination patterns.^19^ In addition, quantum mechanical methods are applied to accurately model metalloproteins but at much more expensive cost.^20^ Density functional theory (DFT) methods are also widely used to model zinc with molecules like amino acids and peptide fragments.^21,22^ A comprehensive review on zinc and other transition metal ions has been published by Li and Merz.^23^

Recently, building a potential energy surface (PES) via machine learning (ML) using high-level quantum chemistry methods as reference data has increasingly gained attention in computational chemistry.^24−27^ Well-built machine-learning potentials (MLPs) can achieve high accuracy as their reference method but at a much lower computational cost. ML models have wide applications in organic molecules probably because of the relatively simple electronic structures and bond types. And there are already some benchmark sets available, which includes various organic molecules, such as QM9,^28^ MD17,^29^ GDB-13,^30^ and ANI-1.^31^ Neural network potentials (NNPs) trained on such data sets of small molecules already reach ∼0.1 kcal/mol error.^32^ However, ML models for transition metal complexes (TMCs) have not been as widely investigated.^33−35^ A main challenge to the accurate modeling of these TMCs is the scarcity of sufficient geometries to cover their potential energy surfaces. There are already a few publicly available data sets for TMCs,^36−38^ but none of them considers multiple conformers. The process of conformational sampling is important in determining the physicochemical and macroscopic properties of molecules, as the three-dimensional (3D) arrangement of atoms strongly influences various properties.^39^ Moreover, molecular conformations can affect the biological activity as well. For example, DNA only binds to a zinc transcriptional regulator once zinc coordinates in a specific manner and affects a conformational change in the transcription regulator.^40^

In this work, we first create a data set, which includes both configurationally and conformationally diverse zinc(II) coordination geometries using metadynamics (MetaMD). With this well-curated data set, we then use ML techniques to model zinc complexes.

## Methods

### Data Set

**Figure 1:**
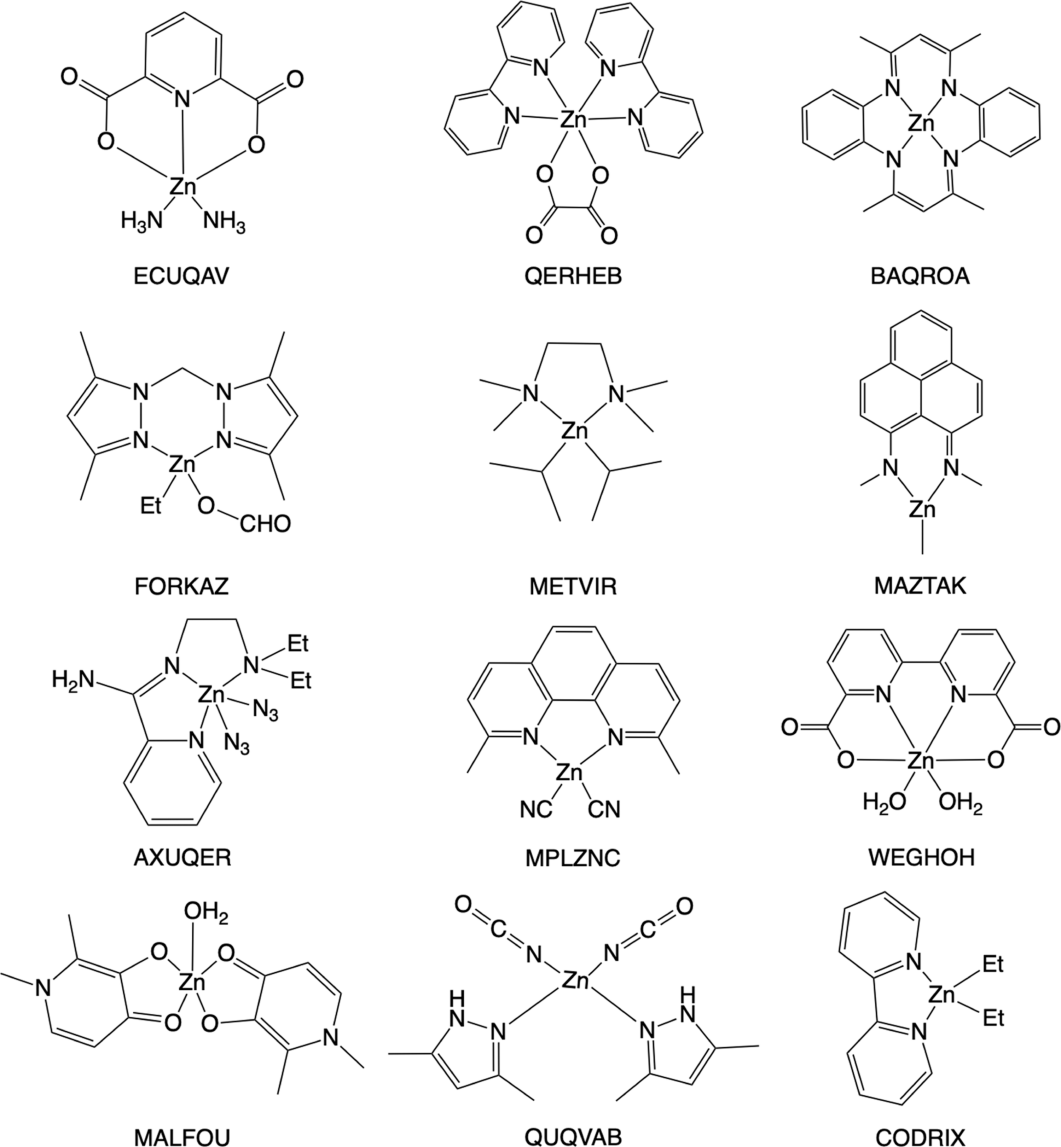
Some structures of zinc complexes with the refcode taken from the CSD.

**Figure 2:**
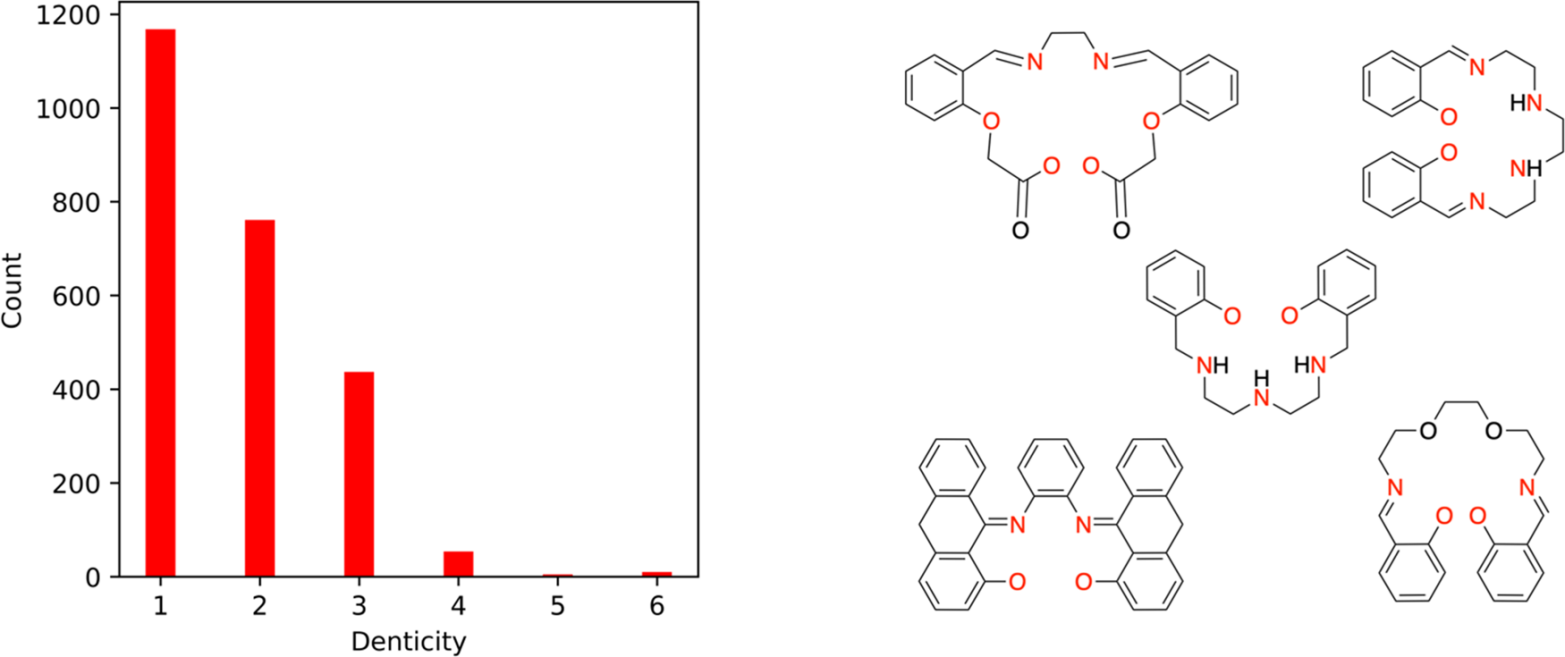
Denticity distribution of ligands in 771 complexes and representative examples of the polydentate ligand. The coordinating atoms are highlighted in red.

**Figure 3:**
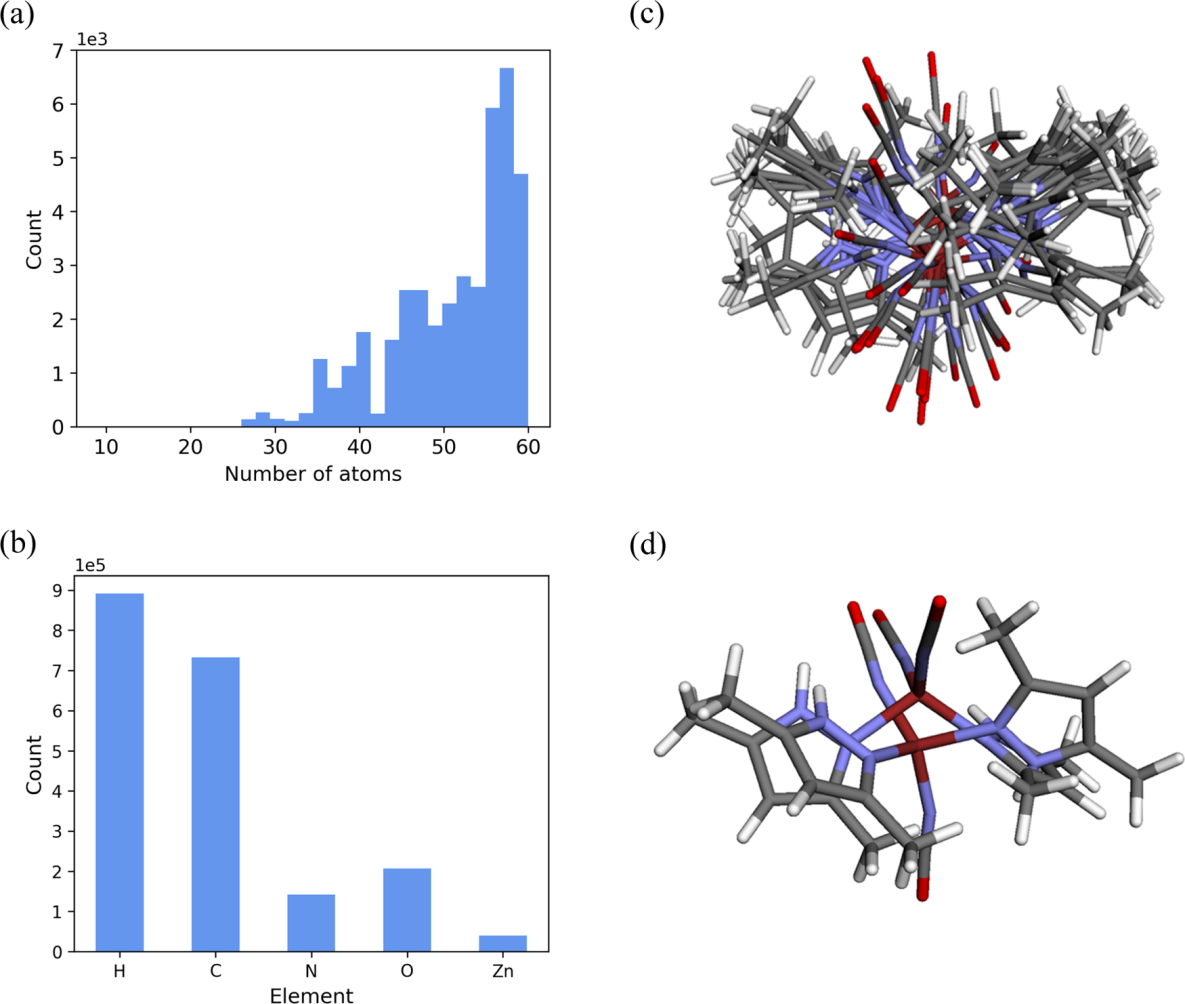
Chemical space of the zinc_60 data set. (a) Molecular size distribution. (b) The distribution of H, C, N, and Zn. (c) An ensemble example of 19 conformers of refcode: QUQVAB. (d) The geometries of the highest energy and the lowest energy in the ensemble, Δ*E* = −5.76 kcal/mol, RMSD = 2.47 Å.

The transition metal quantum mechanics (tmQM) data set,^36^ consisting of a collection of GFN2-xTB^41^-optimized TMCs from the Cambridge Structural Database (CSD),^42^ is used as the base data set. From the tmQM data set, we extracted 771 complexes of 60 atoms or less, each of which includes H, C, N, O, and Zn. All complexes are mononuclear, closed-shell, and neutral. Some examples are given in Figure 1. Various bonding patterns and ligand types are present in this data set. As shown in Table 1, the coordination number of these 771 complexes ranges from 2 to 8, leading to 38 total denticity types. And 2435 ligands were identified from the ensemble of complexes, of which 829 ligands are unique (evaluated by SMILES). The denticity distribution of the 2435 ligands is given in Figure 2. To build the PES, multiple conformations of each molecule were generated using metadynamics (MetaMD),^43^ which has been shown to be a good sampling method in NNP research.^44,45^ The canonical molecular dynamics (MD) simulation is a typical way to prepare a data set for NNPs. However, this is an energetically uneven sampling method, i.e., most geometries are generated in low-energy regions, whereas configurations with relatively high energy are rarely visited due to energy barriers. Such a data set generation method may result in a biased neural network, which can reach high accuracy in dense configuration regions, but for high-energy configurations, large errors can occur. This limitation can be overcome by using MetaMD, which introduces extra potentials against previously generated geometries to encourage frequent sampling in high-energy regions. In this work, we used CREST^46^ to automatically explore conformation space of zinc complexes. In CREST, the biased potential is applied as the summation of multiple Gaussian functions in terms of the root-mean-square deviation (RMSD)
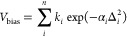
1where *n* is the number of all reference structures, the RMSD related to the *i*th reference structure is utilized as the collective variable Δ_*i*_, *k*_*i*_ is the pushing strength, which can be scaled by the size of the structure, and the parameter α_*i*_ determines the width of the potential. A dozen MetaMD simulations with different combinations of *k*_*i*_ and α_*i*_ were performed in parallel to obtain a complete conformation ensemble. From the 771 zinc complexes, 53 247 conformations were obtained through CREST. Highly overlapping conformations were removed after RMSD calculation^47^ between each pair with a cutoff of 0.1 Å. Each pair was aligned to get the true minimal RMSD. The single-point energies were then calculated at the meta-GGA r^2^SCAN-3c^48^ level, a composite electronic-structure method. All calculations were conducted using ORCA 5.0.4,^49^ with *TightSCF* and all other parameters set to the default. Eventually, 39 599 conformations comprised the zinc_60 data set (Figure 3), which is randomly split into training (31 601), validation (3999), and test set (3999).

**Table 1:** Coordination Types in the 771 Complexes

CN^a^	denticity type	count	CN	denticity type	count
2	1,1	25	6	1,1,1,3	10
3	1,1,1	3		1,1,4	4
	1,2	18		1,1,2,2	134
	3	1		1,2,3	14
4	1,1,1,1	27		1,5	3
	1,1,2	23		2,2,2	27
	1,3	21		2,4	3
	2,2	66		3,3	41
	4	17		6	10
5	1,1,1,1,1	1	7	1,1,1,1,3	4
	1,1,1,2	8		1,1,2,3	14
	1,1,3	28		1,3,3	10
	1,4	27		2,2,3	20
	1,2,2	42		4,3	2
	2,3	8	8	1,1,3,3	52
	5	2		1,3,4	1
6	1,1,1,1,1,1	40		2,2,2,2	1
	1,1,1,1,2	10		2,3,3	54

a CN: coordination number.

### Neural Network

A widely used NNP model is called a “message passing neural network” (MPNN) proposed in 2017,^50^ which is a framework of graph neural networks (GNNs), in which each object is regarded as a graph consisting of node, edge, and global attributes. The graph manifests the relations (edges) among a collection of nodes. The information on the graph is embedded into its nodes and edges, and these embeddings are progressively processed without changing the connectivity of the graph. In terms of NNPs, each molecule is a graph, where atoms represent nodes, and bonds are encoded into edges. Once the representation of each node is randomly initialized, each node will go to the interaction block, which includes message passing and update steps. In the (*t* + 1)th block, the neighbors’ messages of the *t*th block *h*_*j*_^*t*^ are collected via *M*_*t*_ functions
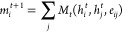
2where *m*_*i*_^*t *+ 1^ is the total message passed from the neighbors to the central node and the bonded information on pairwise atoms *e*_*ij*_ is considered to accurately describe the local atomic environment of the central node. The updated representation of the center node *h*_*i*_^*t* + 1^ depends on both its own representation *h*_*i*_^*t*^ and the message passed from the neighbors *m*_*j*_^*t* + 1^
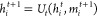
3where *U*_*t*_ is a nonlinear function designed by the neural network. Each node goes through both *M*_*t*_ and *U*_*t*_, which is called one interaction. And these interactions are usually iterated a couple of times, so that the message can propagate in the molecule. Each node then does the atom-wise transformations to convert its final embeddings *h*_*i*_^*T*^ into the atomic energy *E*_*i*_ via *R* functions in the read-out stage
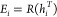
4

Finally, all atomic energies are summed as the total energy of a given chemical system. A well-designed example of an MPNN is the SchNet^51^ model, where the continuous filter convolution is used to model the message passing between each pair of atoms.

Many variants of SchNet have been proposed in recent years like PAINN,^32^ NequIP,^52^ tensor field networks (TFNs),^53^ SE(3) transformers,^54^ to name a few. Such equivariant graph neural networks (EGNNs) can leverage the symmetry of the data by operating equivariant transformations on 3D molecules. But one limitation that all of these models have is the neglect of long-range interactions. To simplify the computation, these models only consider local atomic embeddings, where the feature of each atom is initialized based on its atom type. And only the neighbors whose relative position is within a predefined cutoff are considered to model the pairwise short-range interactions by using some nonlinear functions. These short-range interactions are then used to update the atomic embedding, which predicts the atomic energy at the final step in eq 4. Since the interactions are limited to a certain range, the long-range interactions are greatly ignored.^55^

To circumvent this limitation, a typical way is to include these long-range interactions explicitly by using standard physical forms like PhysNet,^56^ SpookyNet,^57^ and 4G-HDNNPs.^58^ In these efforts, to model these long-range interactions, the first step is to predict the partial charges using neural networks, and then the electrostatic interactions are calculated using Columb’s law. The dispersion corrections can also be included by following methods like DFT-D3.^59^ Since the D3 correction method uses predefined equations with pretrained universal parameters for all systems, large errors may appear for some specific systems. To model the long-range interactions more accurately, a better choice is to incorporate the long-range interactions implicitly by using a neural network. Recently, EwaldMP^60^ and So3krates^61^ have shown good results in modeling these nonlocal interactions for organic molecules^62,63^ and periodic materials.^64^

**Figure 4:**
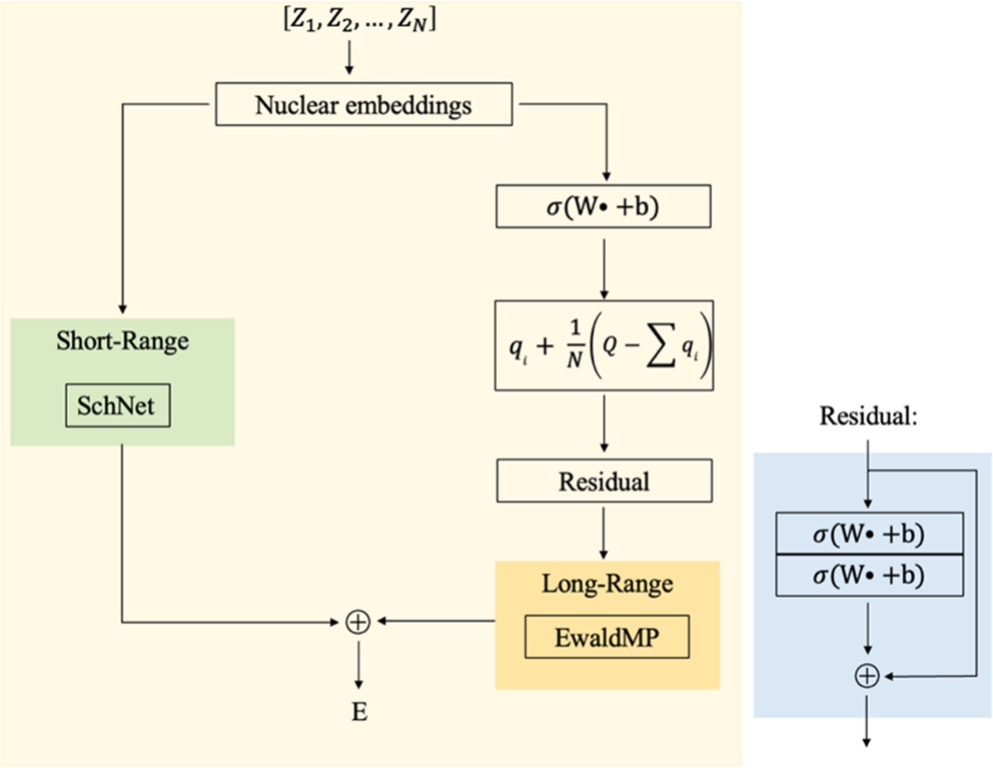
Schematic model used in this work.

EwaldMP is an Ewald-based message passing block inspired by the Ewald summation.^65^ It is important to realize that within a neural network, the Ewald block can represent both long-range electrostatic interactions as well as long-range dispersion interactions through the process of modeling over a data set, such as ours, that has both types of interactions. In EwaldMP, the features for long-range interactions in real space are transformed into Fourier space. Such transformations enable the frequency to decay quickly and rapidly converge at a threshold value. As a result, the long-range interactions can be summed up efficiently. EwaldMP is a standalone block that can be added to any short-range model. In the original EwaldMP work, the same nuclear embeddings are fed into both the baseline model and EwaldMP. Considering the importance of partial charges in modeling these long-range interactions,^66,67^ here, we propose a simple but efficient way to incorporate the partial charges into EwaldMP to better model the long-range interactions of zinc complexes. The inputs of the model are the Cartesian coordinates *r*_*i*_ ∈ *R*^3^, the total charge of complex *Q* ∈ *Z*, and the atom types, which are decoded by the nuclear charge *Z*_*i*_ ∈ *N*. These nuclear charges are projected to a high-dimensional space by looking up an embedding table to form the nuclear embeddings *x*^0^ ∈ *R*^*F*^, where *F* is the number of features. Instead of feeding the same nuclear embeddings into both baseline and EwaldMP models, in this work, the nuclear embeddings are further transformed into partial charges via nonlinear functions defined by a neural network


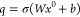
5where σ is the activation function and *W* and *b* are trainable parameters. To better model the electronic structures, these partial charge embeddings are scaled to make sure that the sum of these partial charges is equal to the total charge *Q* via
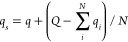
6
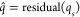
7where a two-layer residual block is used to avoid gradients vanishing or exploding.^68^ These predicted partial charges are then fed into EwaldMP to model long-range interactions. The schematic process is given in Figure 4.

### Computational Procedure

To test the performance of the partial charge embedding on modeling the long-range interactions, we run three types of models on the zinc_60 data set: (i) Baseline. These models cover only the short-range interactions. The baseline models we used in this work are SchNet and PAINN and the input of the baseline model is only the nuclear embedding as proposed in the original work: (ii) Baseline+EwaldMP. The EwaldMP model is used to model the long-range interactions. In this case, both baseline and EwaldMP models share the same nuclear embedding, which is the approach used in the original EwaldMP work: (iii) Baseline + EwaldMP_Q. The nuclear embedding is still fed into the baseline model, while the EwaldMP model now accepts the partial charge embedding, i.e., EwaldMP_Q, as illustrated in Figure 4.

All models used the same initial learning rate of 5 × 10^–4^. The SchNet-related models are trained with a batch size of 64, and a milestone scheduler sets 30,000 steps to warm up at a warmup factor of 0.2 and then decays at 60,000, 90,000, and 120,000 steps with a decay factor of 0.1, while the PAINN-related models use the Adam optimizer, with the plateau scheduler (with a patience of 10 and a decay factor of 0.5), and the batch size is 32.

We also compared our proposed model with some widely used semiempirical methods, i.e., GFNn-xTB (*N* = 1, 2),^69,70^ PM6-D3H4X, and PM7.^71−73^ To do this, we extracted 3838 conformations from the test data set. We did not use the whole test data set since some complexes in the test data set only have one conformation, which is not applicable to compare the relative energies among different computational methods. GFNn-xtb calculations were conducted in the *xtb*(74) package (version 6.6.1). The PM6-D3H4 and PM7 calculations were performed using version 22.0.6 of the MOPAC program.^75,76^ We then calculated the conformational energies at the double-hybrid PWPB9576/CBS (def2-TZVPP/def2-QZVPP) level with the D4 correction based off its known accuracy for TMCs.^77^ These calculations were performed in ORCA 5.0.4^49^ with *TightSCF* and the RIJK approximation.^78^

## Results and Discussion

We first compared the performance of three types of models. The total energy of each geometry in all 3999 conformations of the test set was predicted by each model, and the results are given in Table 2. Our proposed model (baseline + EwaldMP_Q) reaches the lowest error in both cases, decreasing the error by 23 and 25% relative to the SchNet and PAINN baseline model, respectively. The results indicate that long-range interactions are important in accurately modeling zinc complexes. Since the long-range interactions are highly dependent on the atomic partial charges,^79^ accurate partial charge embeddings are of great importance for modeling the long-range interactions. The original EwaldMP model, which takes the nuclear embeddings as inputs, only considers the representations of single atoms. It can only guarantee that the atoms of the same type share the same embeddings, while the global charge attribute is ignored. Instead, in our proposed EwaldMP_Q scheme, the nuclear embeddings are further transformed and scaled to get the partial charge embeddings to make sure that the sum of such embeddings matches the total charge of the whole system. The results validate our hypothesis. The original EwaldMP model yields the largest errors, which are 2.42 and 1.50 kcal/mol, increasing the error by 101 and 10% from those of the baseline models, respectively.

**Table 2:** Mean Absolute Error (MAE) for the Total Energy Predictions in kcal/mol with Reference to r^2^SCAN-3c^a^

model	MAE
SchNet	1.20
SchNet + EwaldMP	2.42
SchNet + EwaldMP_Q	**0.92**
PAINN	1.36
PAINN + EwaldMP	1.50
PAINN + EwaldMP_Q	1.02

a Best result in bold.

**Figure 5:**
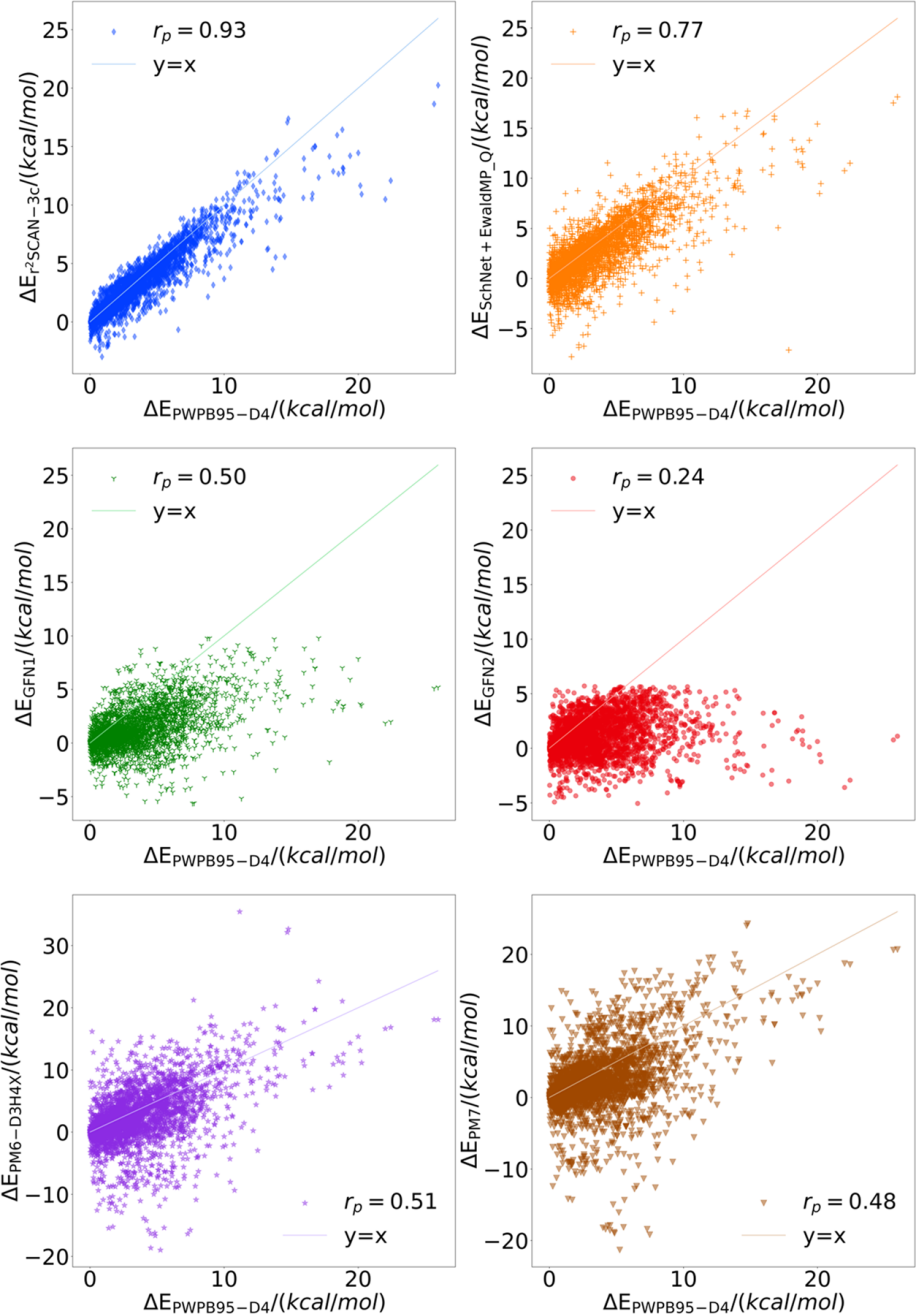
Relative conformational energies in all tested methods with reference to the PWPB95-D4 method.

To investigate the performance of the semiempirical methods on the zinc_60 data set, we compared the MAE of each tested method in reference to the PWPB95-D4 method. As shown in Table 3, our proposed model clearly outperforms the semiempirical methods with an MAE value of 1.32 kcal/mol, but it performs worse than the composite r^2^SCAN-3c method, which is the reference method used to train the model. The overall trends of these relative conformational energies are listed in Figure 5. The reference PWPB95-D4 method is along the *x*-axis, while various tested methods are along the *y*-axis. The Pearson correlation coefficient (*r*_p_) is also reported. In the top left panel, the correlation between PWPB95-D4 and r^2^SCAN-3c is shown and, not unexpectedly, it is generally good with an MAE of 0.65 kcal/mol, which indicates this method’s reliability for curating the reference data of NNPs. And our ML method follows a trend similar to that in Figure 5.

**Table 3:** Performance of All Tested Methods on 3838 Conformations with Respect to the PWPB95-D4/CBS Method^a^

method	MAE	count
r^2^SCAN-3c	0.65	150
SchNet + EwaldMP_Q	1.32	529
GFN1-xTB	2.15	764
GFN2-xTB	2.35	970
PM6-D3H4X	2.39	796
PM7	2.41	782

a The mean absolute errors (MAEs) are given in kcal/mol. The count is the number of conformations, which have lower energy than the actual most stable conformation, i.e., the lowest energy conformation at the PWPB95-D4 level.

We also did some qualitative analysis. The 3838 conformations can be grouped into 418 ensembles, each of which has at least two conformations but in the same configuration. We then ranked the conformations in each ensemble by energy. In each ensemble, the lowest energy conformation at the PWPB95-D4 level is regarded as the most stable conformation. We then count the number of conformations, which is ranked more stable than the reference geometry, i.e., the geometry has lower energy than the reference geometry evaluated by each tested method. This analysis indicates the possibility of each tested method mistakenly locating the most stable conformation. As shown in Table 3, for the r^2^SCAN-3c method, only 150 conformations, i.e., the number of *y*-axis values below 0 in Figure 5, are incorrectly predicted to have lower energy than the actual lowest energy conformation at the PWPB95-D4 level, and the number of such cases in our SchNet + EwaldMP_Q method is 529. However, in the semiempirical methods, the MAE is larger than 2 kcal/mol, and the number of incorrect low-energy conformations is more than 700, among which PM7 yields the largest MAE of 2.41 kcal/mol, while GFN2-xtb yields the largest number of incorrect low-energy conformations (970 out of 3838 conformations). Interestingly, the GFNn-xtb method has a bias to lower the relative energies; as shown in Figure 5, the *y*-axis range in GFNn-xtb is around 10 kcal/mol, while the reference relative energy range, i.e., the *x*-axis range is around 25 kcal/mol, but other tested methods generally match this range well. Overall, the results indicate that our ML method outperforms these semiempirical methods in both qualitative and quantitative evaluations.

Finally, our proposed ML method can greatly reduce the computation cost. It only took 10 s to predict these 3999 conformations on a single GPU, while the average time of r^2^SCAN-3c in our test set was 4.46 min running on 4 central processing unit (CPU) processors for each geometry. For the PM6-D3H4X method, the average time was 0.97 s, while it took 0.94 s for the PM7 method. And the GFN1-xtb method took 6.65 s on average, while GFN2-xtb took 10.82 s for each geometry. All semiempirical methods used only 1 CPU processor. Unfortunately, given the difference in processors used between the ML approach (GPU) and the other approaches (CPU), it is not possible to make a quantitative comparison.

## Conclusions

Building the PES of TMCs is still a significant challenge because of their unique, but complicated, electronic structures. And the lack of a synthetic data set composed of tens of thousands of geometries poses an additional obstacle. In this work, we first curated a zinc(II)-ligand complex data set, which includes conformational energies. These conformations were generated by using metadynamics supported by CREST to cover broad energy regions. With this data set, we then built NNPs for these zinc complexes. To better cover the long-range interactions, we incorporate partial charges into the Ewald message passing model, which reaches the lowest mean absolute error of 0.92 kcal/mol with reference to the r^2^SCAN-3c method, significantly decreasing the error from the baseline models and the originally proposed Ewald message passing method. The performance of this model also outperforms several semiempirical methods but at a reduced cost. In the future, more advanced charge schemes can be used to more accurately predict the partial charges that are then input into the Ewald message to better model long-range interactions.

## Data Availability

All data and code are available at https://github.com/Neon8988/Zinc_NNPs.

## Supporting Information Available

The curated CSD complexes used to generate conformations in this work (XLSX)
The semiempirical level energy of all geometries used for energy comparison (XLSX)
